# Diagnosing a rare thoracic intramedullary spinal dermoid cyst using DWI with ADC mapping: Case report

**DOI:** 10.1016/j.amsu.2022.104440

**Published:** 2022-09-02

**Authors:** Jennifer P. Adams, Daniel Habenicht, Sohum K. Desai

**Affiliations:** a University of Texas Rio Grande Valley School of Medicine, Harlingen, TX, USA; b Department of Surgery, University of Texas Rio Grande Valley School of Medicine, Harlingen, TX, USA

**Keywords:** Dermoid cyst, Intramedullary spine tumor, Laminectomy

## Abstract

**Introduction:**

Dermoid cysts are rare lesions generally associated with embryological errors that occur during neural tube closure. Intramedullary lesions are extremely rare, especially within the upper thoracic spinal cord.

**Case presentation:**

We report a case of a 19-year-old male who had an intramedullary thoracic dermoid cyst presenting with progressive ataxia, lower limb weakness, and hyperreflexia. MRI demonstrated a 1.2 × 1.8-cm intramedullary thoracic dermoid cyst causing significant spinal cord compression, which was successfully removed via full resection. The patient had an uncomplicated postoperative course, with improvement in preoperative deficits.

**Discussion:**

This is a unique case documenting a thoracic spinal cord intramedullary dermoid cyst not associated with trauma or congenital abnormality of the spinal cord.

**Conclusion:**

We highlight the importance of future inclusion of diffusion-weighted magnetic resonance (MR) imaging (DWI) with apparent diffusion coefficient (ADC), an imaging modality that detects differences in cellularity of spinal cord lesions, for earlier diagnosis of dermoid cyst.

## Highlights

• This is a unique case of intramedullary dermoid cyst not associated with trauma or congenital abnormality.
• Diffusion-weighted magnetic resonance (MR) imaging (DWI) with apparent diffusion coefficient (ADC) was used to detect differences in cellularity of the spinal cord lesion.
• We recommend future use of DWI and ADC mapping for early detection of intramedullary neoplasms.

## 1 Introduction

Intramedullary spinal dermoid cysts are rare and insidious tumors that occur most commonly due to ineffective closure of the neural tube during the development process [1,2]. They are slow-growing tumors that are clinically detected when neurologic symptoms manifest between the second and third decades of life. The prevalence of intramedullary dermoid cysts is 1% worldwide. Economic impact and patient satisfaction in regaining quality of daily living for individual patients after surgical decompression is substantial. Advances in neurosurgery have made dermoid cyst removal faster and safer with excellent perioperative recovery.

This work has been reported in line with the SCARE 2020 criteria [11].

## 2 Case Presentation

**Fig. 1:**
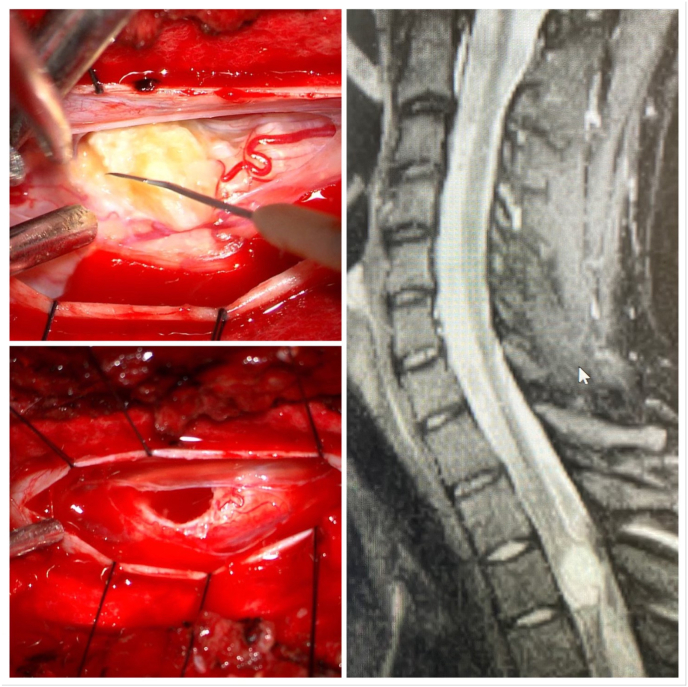
Intraoperative photographs showing an exophitic, yellowish, pearly tumor emanating from the right side of spinal cord at T3 (Left, Above). The cavity of the dermoid cyst after complete resection (Left, Bottom). Sagittal T1-weighted image revealed a well-encapsulated hypointense lesion within the spinal canal resulting in cord compression (Right).

**Fig. 2:**
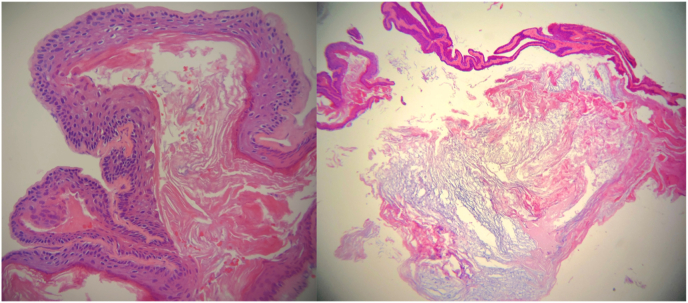
Histopathology with Hematoxylin and eosin staining demonstrates cyst wall lined with stratified squamous epithelium and lumen containing loose keratin and mature skin appendages consistent with dermoid cyst.

We present a 19 year old male with two month history of ataxia secondary to an intradural intramedullary expansile dermoid cyst in the spinal cord. The patient reported to clinic with progressively worsening numbness of the lower extremities and with difficulty walking beginning three months ago. Physical exam showed 2/5 strength in right lower extremity and 3/5 motor strength in left lower extremity, spastic with exaggerated reflexes and bilateral positive Babinski signs. There was no history of incontinence, infection, spinal trauma or previous spinal surgery. The overlying skin of the thoracolumbar region was intact, showing no signs of inflammation, nevi, or local hair growth. Patient denied history of weight loss, night sweats, body aches. Family history was noncontributory. The patient underwent a lumbar puncture which demonstrated elevated protein count but was otherwise negative. MRI scan of the dorsal spine was performed with T1-and T2-weighted images, which evidenced a focal, well-defined space-occupying lesion measuring 3.5 × 3.8cm [2]. T1-weighted image revealed a well-encapsulated hypointense lesion (Fig. 1) within the spinal canal. Prior to surgery, patient was placed under general anesthesia in prone position. Injection of methylprednisolone was given intraoperatively and continued in the postoperative period at a dose of 25 mg/kg bolus over 1 hour followed by 5 mg/kg/hr for 23 hours. The patient underwent standard posterior laminectomies and fenestration of T2, T3 and T4 performed by the in-house neurosurgeon. Intraoperative ultrasound was performed to examine the spinal cord and revealed tumor of complex shape with loculations located around T3-4. A midline myelotomy was performed and tissue was resected for the purposes of decompression and biopsy procedures. Upon opening of the arachnoid, an exophitic, yellowish, pearly tumor emanating from the right side of spinal cord at T3 was visualized and fully excised (Fig. 1). Examination of the specimen revealed a 1.2 × 1.8cm cyst filled with a soft, yellow waxy substance with glandular secretions and areas of induration. Histopathology exam confirmed the diagnosis of dermoid cyst. Histopathology appearance demonstrated cyst wall lined with stratified squamous epithelium and lumen containing loose keratin and mature skin appendages (Fig. 2).

The patient had an uncomplicated postoperative course without abnormal neurological sequelae. At the 6-month follow-up, he reported improvement in all of his preoperative deficits including right lower extremity strength and ambulation. Follow-up radiological evaluation showed no evidence of disease recurrence.

## 3 Discussion

Intraspinal dermoid cysts are exceptionally rare and insidious tumors that account for less than 1% of all spinal tumors [1,2]. They are known to have a predilection for the lumbosacral spine 60%, thoracic spine 10% and cervical spine 5% [3,4]. Such cysts are diagnosed when patients present with neurologic manifestations, including paresthesias, paralysis and sphincter complications [5]. Bladder or bowel incontinence is most common amongst previously reported cases in the literature [3]. In this case, the patient presented weakness and paresis along with progressive ataxia, which is an uncommon symptom.

Inclusion dermoid cysts are classified as either congenital or acquired. From a congenital developmental perspective, dermoids arise from cells retained from defective closure of the neural tube between the third and fourth week of embryonic life [6]. Many of the intraspinal thoracolumbar dermoid cysts that arise spontaneously are seen in association with other congenital anomalies of the spinal cord, vertebrae, and the soft tissues overlying them dorsally [7].

Acquired dermoid cysts most commonly arise secondary to trauma from spinal instrumentation or iatrogenic injury. Acquired dermoids have been documented in children who have undergone multiple lumbar punctures for treatment of tuberculous meningitis and after myelomeningocele repair [7]. Acquired dermoids occur years after the lumbar punctures and are thought to result from penetration of skin fragments [7]. Our patient denied any history of congenital spinal defects or history of invasive spinal procedures. A similar case described a 14 year-old male without history of congenital disorders presenting with an intraspinal intramedullary cyst [8]. The cyst contained an abscess at the level of T12 and L1, causing localized cord compression. In contrast, our patient did not report any symptoms of bowel or bladder incontinence. Our case gives a unique clinical picture of a spinal intramedullary dermoid cyst without history of spinal dysraphism or symptoms of incontinence.

We suggest future use of diffusion-weighted magnetic resonance (MR) imaging (DWI) with apparent diffusion coefficient (ADC), which reflects tissue cellular capacity and helps with early detection of lesions such as intramedullary neoplasms and demyelinating diseases to assess the feasibility of surgical resection, medical treatment, and follow-up [9]. Conventional MRI has had limitations in differentiating lesions in the region of the spinal cord. Vadivelu et. Al reported two pediatric cases of previously undiagnosed lumbar dermal sinus tracts. One terminated in an infected dermoid cyst and intramedullary abscess and the other terminated in holocord edema. Standard pre-operative magnetic resonance imaging (MRI) studies in these patients were initially confused for an intramedullary spinal cord tumor [10].

The DWI technique is based on the principle that regulate free water motion in cellular environments. The calculation of ADC provides quantitative measurement of molecular water movement [9]. Hypercellular tumors, metastases and fibrosis that have increased cellularity and restricted free extracellular space will show impeded water diffusion compared with normal surrounding tissues and thus higher DWI signal intensity and low ADC values. Abscess and necrosis have disrupted cell membranes and less restriction of water diffusion, translating into lower DWI signal intensity and higher ADC values [9]. Preoperative DWI may provide a more detailed information of the proximity of the dermal cyst to surrounding nervous tissue, which offers insight into whether complete resection offers an advantage over partial resection in improved long-term outcome.

The treatment of choice for dermoid tumor is the total excision of the mass at an early stage, which often difficult to achieve since the capsule adheres to the cord [5]. Magnetic resonance imaging for diagnosis and surgical decompression of the dermoid cyst carried out by standard micro neurosurgical technique employed for other intramedullary tumors is the mainstay of management. Future studies should be undertaken to assess the role of DWI in the diagnosis and differentiation of intramedullary spinal lesions.

## 4 Conclusion

True intramedullary dermoid cysts are rare, especially in a thoracic or cervical location. Their etiology is likely developmental or iatrogenic, with most cases associated with congenital lesions such as spina bifida and dermal sinus tracts. We presented a patient with a mid-thoracic intramedullary dermoid cyst without history of trauma or congenital spinal lesions. Intramedullary dermoid cysts should be considered in the differential in a young person with progressive neurological symptoms. We support the potential value of DWI imaging with ADC mapping in early diagnosis of dermoid tumor and preoperative surgical planning.

## Ethical approval

Not applicable.

## Sources of funding

None.

## Author contribution

JA was involved in the writing of the manuscript. SKD was involved in the editing/supervision of the manuscript.

## Registration of research studies

1 Name of the registry: N/A
2. Unique identifying number or registration ID: N/A
3. Hyperlink to your specific registration (must be publicly accessible and will be checked): N/A

## Guarantor

Sohum K. Desai MD.

## Informed consent

Written informed consent was obtained from the patient for publication of this case report and accompanying images. A copy of the written consent is available for review by the Editor-in-Chief of this journal on request.

## Provenance and peer review

Not commissioned, externally peer-reviewed.

## Patient perspective

N/A.

## Declaration of competing interest

All authors declare no conflicts of interest.
